# Testicular seminoma in patients receiving adalimumab: two case reports^⋆^

**DOI:** 10.1016/j.abd.2026.501317

**Published:** 2026-03-26

**Authors:** Beatriz Figueira Vilela, Inês Aparício Martins, Nélia Cunha, Joana Cabete

**Affiliations:** Department of Dermatovenereology, Santo António dos Capuchos Hospital, Unidade Local de Saúde de São José, Lisbon, Portugal

Dear editor,

Anti-Tumor Necrosis Factor alpha (TNF-α) agents, particularly adalimumab, are widely used in dermatology to manage inflammatory conditions such as psoriasis and hidradenitis suppurativa. While chronic immunosuppression is known to increase malignancy risk due to impaired immune surveillance, TNF-α inhibitors have not been associated with an overall increase in cancer incidence, aside from non-melanoma skin cancers and hematologic malignancies.^1^ Reports of solid tumors, including testicular cancer, remain rare and poorly understood. This article presents two cases of patients who developed testicular seminomas following treatment with adalimumab. The aim of this article is to highlight this potential association and emphasize the importance of vigilant oncological monitoring in patients receiving long-term biologic therapy.

A 36-year-old male with severe inflammatory hidradenitis suppurativa (iHS4 > 10, Hurley stage III), presenting with axillary, inguinal and genital fistules, was diagnosed with testicular seminoma 4-years and 9-months after initiating treatment with adalimumab (80 mg every other week). The patient reported progressive right-sided testicular swelling and pain, and a firm mass was palpated. Following urological evaluation, a right inguinal orchiectomy was performed, and histopathology confirmed a testicular seminoma (pT1 N1 M0). Adjuvant chemotherapy with cisplatin, bleomycin, and etoposide was administered. After discussion with the assisting oncologist, adalimumab was discontinued. Two years after the diagnosis, the patient remains free of seminoma recurrence.

A 33-year-old male with severe nail and arthropathic psoriasis, as well as mild hidradenitis suppurativa in the axillary and gluteal regions, was diagnosed with testicular seminoma 11-months after initiating treatment with adalimumab (40 mg every other week). The patient presented with back pain, night sweats, asthenia, and significant weight loss (8 kg in one month, approximately 10% body weight). Laboratory workup revealed leukocytosis with neutrophilia, an elevated C-reactive protein of 128 mg/L, and hematuria (8784 RBCs/mL). Given the immunosuppressed state, a thoraco-abdomino-pelvic CT scan was performed, revealing a large left-sided adenopathic conglomerate causing left hydronephrosis. A scrotal ultrasound identified a solid intratesticular nodule. A left inguinal orchiectomy was performed, and histopathological analysis confirmed a testicular seminoma (pT1 cN3 M0). Following discussion with the treating oncologist, adalimumab therapy was continued. The patient underwent adjuvant chemotherapy with cisplatin, bleomycin, and etoposide. However, eight weeks after completing chemotherapy, disease progression was observed, and the patient ultimately succumbed to the disease.

Although a direct causal relationship cannot be established, these cases raise the possibility of a rare but biologically plausible association between adalimumab and testicular seminoma. Both patients were within the typical age range for this malignancy (25- and 40-years), yet additional clinical features may have contributed. While large meta-analyses have not demonstrated a significant increase in overall malignancy risk associated with TNF-α inhibitors, rare tumors may be underreported due to short follow-up and limited reporting.^1^ Notably, a previous report described a mixed germ cell tumor following infliximab therapy,^2^ and experimental studies have shown that TNF-α promotes germ cell apoptosis and may act as a tumor suppressor in testicular tissue.^3^, ^4^ Its pharmacological inhibition could, therefore, compromise local immune surveillance and facilitate germ cell proliferation and malignant transformation.

Taken together, these findings reinforce the importance of continued pharmacovigilance and the need to include rare solid organ malignancies in long-term safety monitoring of anti-TNF-α therapies.

## ORCID IDs

Inês Aparício Martins: 0000-0002-2572-2064

Nélia Cunha: 0000-0002-6287-1798

Joana Cabete: 0000-0003-2259-5634

## Authors’ contributions

Beatriz F. Vilela: Design and planning of the study; drafting and editing of the manuscript; data survey, collection, analysis, and interpretation of data; critical review of the literature; approval of the final version of the manuscript.

I. Aparício Martins: Analysis and interpretation of data; intellectual participation in the propaedeutic and/or therapeutic conduct of the studied case; critical review of the literature; approval of the final version of the manuscript.

Nélia Cunha: Analysis and interpretation of data; intellectual participation in the propaedeutic and/or therapeutic conduct of the studied case; critical review of the literature; approval of the final version of the manuscript.

Joana Cabete: Analysis and interpretation of data; intellectual participation in the propaedeutic and/or therapeutic conduct of the studied case; critical review of the literature; approval of the final version of the manuscript.

## Financial support

None declared.

## Research data availability

Does not apply.

## Conflicts of interest

None declared.
